# Cross-Dataset Variability Problem in EEG Decoding With Deep Learning

**DOI:** 10.3389/fnhum.2020.00103

**Published:** 2020-04-21

**Authors:** Lichao Xu, Minpeng Xu, Yufeng Ke, Xingwei An, Shuang Liu, Dong Ming

**Affiliations:** ^1^Academy of Medical Engineering and Translational Medicine, Tianjin University, Tianjin, China; ^2^Department of Biomedical Engineering, College of Precision Instruments and Optoelectronics Engineering, Tianjin University, Tianjin, China

**Keywords:** brain-computer interface, cross-subject variability, cross-dataset variability, deep learning, transfer learning, EEG

## Abstract

Cross-subject variability problems hinder practical usages of Brain-Computer Interfaces. Recently, deep learning has been introduced into the BCI community due to its better generalization and feature representation abilities. However, most studies currently only have validated deep learning models for single datasets, and the generalization ability for other datasets still needs to be further verified. In this paper, we validated deep learning models for eight MI datasets and demonstrated that the cross-dataset variability problem weakened the generalization ability of models. To alleviate the impact of cross-dataset variability, we proposed an online pre-alignment strategy for aligning the EEG distributions of different subjects before training and inference processes. The results of this study show that deep learning models with online pre-alignment strategies could significantly improve the generalization ability across datasets without any additional calibration data.

## 1. Introduction

Brain-Computer Interfaces (BCIs) enable humans to directly control machines via brain signals without any physical intervention (Wolpaw et al., 2002). A typical BCI system consists of three parts: paradigms, neuroimaging techniques, and decoding algorithms. Paradigms are mental tasks that invoke brain activities while the corresponding brain signals are recorded by neuroimaging techniques. Researchers prefer electroencephalography (EEG) among various neuroimaging techniques because of its non-invasive, high temporal resolution, and low-cost characteristics. Decoding algorithms further translate measured brain signals into commands to control computerized devices.

Decoding algorithms are crucial to achieving an efficient and robust BCI system. Over the past 20 years, many effective BCI decoding algorithms have been proposed due to advances in machine learning. Most decoding algorithms extract discriminant features with well-designed spatial filters for improving within-subject classification accuracy. Common Spatial Pattern (CSP) and its variants (Ramoser et al., 2000; Grosse-Wentrup and Buss, 2008; Kai Keng Ang et al., 2008; Lotte and Guan, 2011; Samek et al., 2012) are still most commonly used algorithms for motor imagery (MI) paradigm. For the steady-state visually evoked potential (SSVEP) paradigm, Canonical Correlation Analysis (CCA) (Lin et al., 2007) and Task-related Component Analysis (TRCA) (Nakanishi et al., 2018) are able to improve the speed of SSVEP-based BCI spellers. xDAWN (Rivet et al., 2009) and DCPM (Xu et al., 2018) algorithms are also perform well on P300-based BCI applications. Recently, algorithms based on Riemannian geometry have been introduced into the BCI community, and they provide a unified signal processing framework for decoding brain signals (Barachant et al., 2010; Congedo et al., 2013, 2017a; Lotte et al., 2018). However, most algorithms are unable to reuse pre-trained models across subjects or different sessions of the same subject. These two problems in BCI are referred to as cross-subject and cross-session variability problems. To reduce high variability in individual EEG data, a calibration stage is required to collect training data at the beginning of each session, which is inconvenient for both patients and healthy subjects.

A natural idea for decoding brain signals is to use deep learning models instead of handcrafted feature extraction methods. Manual feature extraction reduces the possibility of utilizing information across subjects. Deep learning utilizes all information in data to train a robust classifier, which often outperforms other machine learning models in classification tasks. Some deep learning models [e.g., Shallow ConvNet (Schirrmeister et al., 2017), EEGNet (Lawhern et al., 2018)] can achieve better performance than traditional methods in within-subject classification task.

Deep learning is also able to ameliorate cross-session and cross-subject variability problems with its robust feature extraction architecture. However, deep learning models used in BCI suffer the lack of data problem. It is hard to collect a sufficient amount of high-quality training data for a specific BCI task. The lack of data problems makes deep learning models easily overfit. Some data augmentation methods may alleviate the overfitting problem for within-subject classification tasks (Wang et al., 2018; Dai et al., 2020). For cross-subject classification tasks, an easier way is to train the model directly on the entire dataset regardless of subject-specific information (Schirrmeister et al., 2017; Lawhern et al., 2018). In practice, however, we found that a pre-trained model from one public dataset may fail to predict new data from another public dataset even if the model performs well on its training dataset. The model is highly specialized in its training dataset structure that a minor change to the test data may make the model invalid. A similar phenomenon was reported in Jayaram and Barachant (2018), where the authors found that the performance of classical supervised BCI algorithms depends on the specific dataset. A public dataset is usually acquired under the same condition in the same lab. Can an algorithm that performs well on one dataset work on another dataset under different conditions? Currently, most studies have only validated the use of deep learning models for a specific dataset, and the generalization ability for other datasets still needs to be further verified. The cross-dataset variability problem in deep learning was proved in our cross-dataset experiment.

In this work, we studied the cross-dataset variability problem of deep learning models. We validated deep learning models across multiple datasets and observed that the optimal model trained for one dataset performs significantly worse on other datasets. The results indicate that deep learning models for BCIs are unable to generalize well outside the training dataset. To alleviate the impact of cross-dataset variability, we introduced an online pre-alignment strategy before the training and validation processes. The results demonstrate that deep learning models with online pre-alignment strategy have better generalization ability across EEG datasets.

## 2. Materials and Methods

### 2.1. Datasets

Eight MI datasets were used in our experiments (Schalk et al., 2004; Leeb et al., 2007; Tangermann et al., 2012; Yi et al., 2014; Zhou et al., 2016; Cho et al., 2017). All datasets are publicly available and details of them are listed in Table 1. CNBCIC2019004 and CBCIC2019004 datasets were downloaded from the 3rd China Brain-computer Interface Competition website. The rest of datasets were downloaded using the MOABB package (Jayaram and Barachant, 2018).

**Table 1:** Details of datasets.

**Dataset**	**Classes**	**Subjects**	**Trials per class**	**Trial duration (s)**	**Channels**	**Sampling rate (Hz)**
BNCI2014001	Left/right/feet/tongue	9	144	4	22	250
BNCI2014004	Left/right	9	360	4.5	3	250
PhysionetMI	Left/right/hands/feet	109	20–30	3	64	250
Cho2017	Left/right	52	100	3	64	512
Weibo2014	Left/right/hands/feet	10	80	4	60	200
Zhou2016	Left/right/feet	4	160	5	14	250
CBCIC2019001	Left/right	18	60	4	59	1000
CBCIC2019004	Left/right	6	40	4	59	250

Three channels (C3, CZ, C4) were used in this work. These channels are located on sensorimotor area and exist in all datasets. Only the left-hand and right-hand MI classes were included in our experiments. Each trial was 3 s in length and downsampled to 100 Hz such that the size of a trial was 3 × 300. All trials were filtered with a 4-order Butterworth bandpass filter of 3–40 Hz. Zero-phase forward and reverse filtering was implemented using filter_data() function in MNE (Gramfort et al., 2013).

For evaluating performance of models, trials were randomly split into training, validation, and test sets. The training set was 80% of the available data. The remaining 20% data were equally partitioned and referred to as validation and test sets. This splitting process was repeated 10 times on each subject, producing 10 different folds.

### 2.2. Notation

In this section, we give the notation and assumptions used throughout the paper. An overview of the notation is listed in Table 2. We assume that the EEG data of each channel is zero mean. This assumption is reasonable in the real world which also widely adopted in many BCI algorithms (Ramoser et al., 2000; Grosse-Wentrup and Buss, 2008). All algorithms below are described with the two-class classification problem in MI paradigm.

**Table 2:** Symbols and notations.

**Symbol**	**Description**
*N*_*t*_	Number of trials
*N*_*c*_	Number of channels
*N*_*s*_	Number of samples
*E*_*i*_	EEG data matrix of a single trial, $E_{i}\in {\mathbb{R}}^{N_{c}\times N_{s}}$
*C*_*i*_	Covariance of *E*_*i*_, $C_{i}\in {\mathbb{R}}^{N_{c}\times N_{c}}$
*W*	Spatial filter matrix, $W\in {\mathbb{R}}^{N_{c}\times L},L\leq N_{c}$
*w*_*i*_	A spatial filter vector, $w_{i}\in {\mathbb{R}}^{N_{c}},W=\left[w_{1},w_{2},\cdots \;,w_{L}\right]$

### 2.3. Traditional Decoding Methods

#### 2.3.1. CSP

The goal of CSP is to find a projection matrix *W* = [*w*_1_, *w*_2_, …, *w*_*L*_], that leads to new time series Ê = *W*^*T*^*E*, which maximizes the discriminance between classes. The CSP algorithm solves the following optimization problem

(1) $$\begin{array}{r} w^{*}=\underset{w_{i}\in {\mathbb{R}}^{N_{c}},i\in \left\{1,2,\cdots \;,L\right\}}{\text{argmax}}\frac{w_{i}^{T}{\bar{C}}^{1}w_{i}}{w_{i}^{T}{\bar{C}}^{2}w_{i}} \end{array}$$

with ${\bar{C}}^{1},{\bar{C}}^{2}$ are average normalized covariance matrices of each class obtained from

(2) $$\begin{array}{r} {\bar{C}}^{k}=\overset{N_{t}^{k}}{\underset{i=1}{\sum }}\frac{E_{i}^{k}{E_{i}^{k}}^{T}}{\text{tr}\left(E_{i}^{k}{E_{i}^{k}}^{T}\right)} \end{array}$$

where $N_{t}^{k}$ is the number of trials of class *k*, *k* ∈ {1, 2} and tr(·) denotes the trace operator. Solutions to (1) are given by eigenvectors of the generalized eigenvalue problem

(3) $$\begin{array}{r} {\bar{C}}^{1}w_{i}=\lambda_{i}{\bar{C}}^{2}w_{i} \end{array}$$

where eigenvalues are sorted in descending order. CSP selects eigenvectors with the *L*/2 largest/smallest eigenvalues to form projection matrix *W*, which is also named spatial filters. The feature vector $f_{i}\in {\mathbb{R}}^{L}$ of *E*_*i*_ is given by

(4) $$\begin{array}{r} f_{i}=\text{log}\left(\frac{\text{var}\left(W^{T}E_{i}\right)}{\sum \text{var}\left(W^{T}E_{i}\right)}\right) \end{array}$$

where var(·) denotes the variance operator on each row of Ê_*i*_ and log(·) denotes the logarithm operator of elements. CSP is usually followed by a linear or non-linear classifier to classify test data.

#### 2.3.2. FBCSP

The Filter Bank Common Spatial Pattern (FBCSP) (Kai Keng Ang et al., 2008) extends the CSP algorithm to EEG data with multiple frequency bands. The goal of FBCSP is to address the problem of manually selecting the subject-specific frequency band for the CSP algorithm. The key step in FBCSP is feature selection, which selects a subset of features that leads to the smallest classification error. FBCSP estimates the importance of each feature vector with mutual information and selects the *L* most important *w* to form the projection matrix *W* used in (4).

#### 2.3.3. MDRM

The Minimum Distance to Riemannian Mean (MDRM) (Barachant et al., 2011) is an algorithm based on Riemannian Geometry. Riemannian Geometry considers matrix *C*_*i*_ as a point in a Riemannian manifold. MDRM computes the Riemannian center of each class and compares Riemannian distances from test points to centers. The Riemannian distance of two covariance matrices *C*_1_, *C*_2_ is given by

(5) $$\delta_{\text{R}}(C_{1},C_{2})=\|\text{Log}(C_{1}^{-1}C_{2}){\|}_{F}={\left[\sum_{i=1}^{N_{c}}{\text{log}}^{2}(\lambda_{i})\right]}^{1/2}$$

where Log(·) is the logarithm operator of a matrix, and λ_*i*_ is the i-th eigenvalue of matrix $C_{1}^{-1}C_{2}$. The Riemannian center ${\bar{C}}_{R}^{k}$ of each class is defined as follows

(6) $$\begin{array}{r} {\bar{C}}_{R}^{k}=\underset{{\bar{C}}_{R}^{k}}{\text{argmin}}\overset{N_{t}^{k}}{\underset{i=1}{\sum }}{\text{δ}}_{\text{R}}\left({\bar{C}}_{R}^{k},C_{i}^{k}\right) \end{array}$$

with *k* ∈ {1, 2}. Although there is no closed form solution to (6) when $N_{t}^{k}>2$, the problem can be solved with iterative algorithms (Moakher, 2005; Pennec et al., 2006; Congedo et al., 2017b). With Riemannian centers, a new test covariance *C*_*test*_ is classified as follows:

(7) $$\begin{array}{r} \underset{k\in \left\{1,2\right\}}{\text{argmin}}{\text{δ}}_{\text{R}}\left({\bar{C}}_{R}^{k},C_{test}\right) \end{array}$$

### 2.4. Deep Learning Models

#### 2.4.1. ShallowNet

ShallowNet (Schirrmeister et al., 2017) imitates FBCSP's design in the deep learning structure. The architecture of ShallowNet is listed in Table 3. The first convolution layer is designed to convolve in a temporal direction, which is analogous to bandpass filtering. The second convolution layer is designed to convolve in a spatial direction, which is analogous to spatial filters in CSP. Shallow ConvNet uses a squaring activation function and average pooling layer to imitate feature mapping in (5). Instead of mutual information selection in FBCSP, ShallowNet uses a fully connected layer to combine all features and predict probabilities of classes.

**Table 3:** ShallowNet architecture.

**Layer**	**Input size**	**Output size**	**Kernels**	**Kernel size**	**Stride**	**Padding**
Conv2d	1 × 3 × 300	10 × 3 × 300	10	(1, 21)	(1, 1)	(0, 10)
BatchNorm2d	10 × 3 × 300	10 × 3 × 300				
Conv2d	10 × 3 × 300	15 × 1 × 300	15	(3, 1)	(1, 1)	(0, 0)
BatchNorm2d	15 × 1 × 300	15 × 1 × 300				
Pow2	15 × 1 × 300	15 × 1 × 300				
AvgPool2d	15 × 1 × 300	15 × 1 × 17		(1, 55)	(1, 15)	(0, 0)
Log	15 × 1 × 17	15 × 1 × 17				
Dropout	15 × 1 × 17	15 × 1 × 17				
Linear	255	2				

#### 2.4.2. EEGNet

EEGNet is a CNN-based model proposed by Lawhern et al. (2018). The architecture of EEGNet is listed in Table 4. EEGNet is designed for general EEG recognition tasks. EEGNet retains temporal and spatial convolution layers in Shallow ConvNet. Instead of simple convolution in ShallowNet, EEGNet introduces depthwise separable convolution (Chollet, 2017) to reduce the number of training parameters. EEGNet also replaces squaring activation with ELU activation.

**Table 4:** EEGNet architecture.

**Layer**	**Input size**	**Output size**	**Kernels**	**Kernel size**	**Stride**	**Padding**
Conv2d	1 × 3 × 300	8 × 3 × 300	8	(1, 31)	(1, 1)	(0, 15)
BatchNorm2d	8 × 3 × 300	8 × 3 × 300				
Depthwise Conv2d	8 × 3 × 300	16 × 1 × 300	16	(3, 1)	(1, 1)	(0, 0)
BatchNorm2d	16 × 1 × 300	16 × 1 × 300				
Elu	16 × 1 × 300	16 × 1 × 300				
AvgPool2d	16 × 1 × 300	16 × 1 × 75		(1, 4)	(1, 4)	(0, 0)
Dropout	16 × 1 × 75	16 × 1 × 75				
Seperable Conv2d	16 × 1 × 75	16 × 1 × 75	16	(1, 15)	(1, 1)	(0, 7)
BatchNorm2d	16 × 1 × 75	16 × 1 × 75				
Elu	16 × 1 × 75	16 × 1 × 75				
AvgPool2d	16 × 1 × 75	16 × 1 × 9		(1, 8)	(1, 8)	(0, 0)
Dropout	16 × 1 × 9	16 × 1 × 9				
Linear	144	2				

### 2.5. Online Pre-alignment Strategy

Recently, many Transfer Learning approaches have been introduced into BCIs to reduce cross-subject variability (Zanini et al., 2018; Rodrigues et al., 2019; Yair et al., 2019). An approach named Riemannian Procrustes Analysis (RPA) was proposed by Rodrigues et al. (2019). RPA takes three steps to match data distributions of source domain and target domain: re-centering, stretching, and rotation. The re-centering step aligns the Riemannian center of covariance matrices to identity matrix. The stretching step modulates dispersions of two domains to the same level. The rotation step further rotates matrices from target domain to match that of source domain with predetermined markers. The re-centering step has also been mentioned in Reuderink et al. (2011) and Zanini et al. (2018) as follows

(8) $$\begin{array}{r} \begin{array}{c} {Ĉ}_{i}=M^{-1/2}C_{i}M^{-1/2} \end{array} \end{array}$$

where *M* is the Riemannian center of training covariances and Ĉ_*i*_ is the aligned covariance matrix. In this work, we applied re-centering step before the training and validation processes, and this is named the pre-alignment strategy (PS). Instead of direct operation on covariances, we transformed *E*_*i*+1_ to aligned time series Ê_*i*+1_ by

(9) $$\begin{array}{r} {Ê}_{i}=\frac{1}{N_{s}}M^{-1/2}E_{i} \end{array}$$

**Figure 1:**
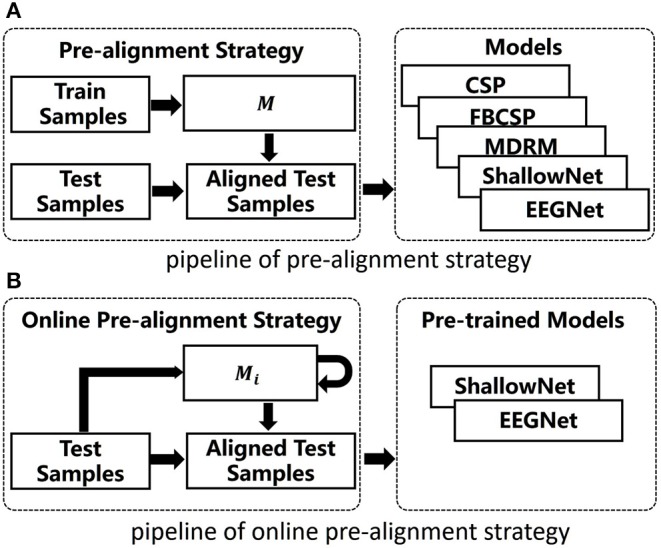
Pipelines of our methods. **(A)** The pipeline of pre-alignment strategy. **(B)** The pipeline of online pre-alignment strategy.

The above transformation has also been mentioned in He and Wu (2020), where the authors used Euclidean mean covariance instead of Riemannian mean covariance *M* here. Although PS is an unsupervised method, they still require enough calibration data of each subject to compute the expected Riemannian center *M*. We implemented an online pre-alignment strategy (OPS) on continuous EEG data for each subject. Assuming that *M*_*i*_ is the Riemannian mean of previous available covariances, *E*_*i*+1_ is the EEG data of the next trial, and *C*_*i*+1_ is the covariance of *E*_*i*+1_. A recursive Riemannian mean update rule is given as follows

(10) $$\begin{array}{r} \begin{array}{c} M_{i+1}=\text{geodesic}\left(M_{i},C_{i+1},\frac{1}{i+1}\right)\\ \;=M_{i}^{1/2}{\left(M_{i}^{-1/2}C_{i+1}M_{i}^{-1/2}\right)}^{\frac{1}{i+1}}M_{i}^{1/2} \end{array} \end{array}$$

where *M*_1_ = *C*_1_. This recursive algorithm was proposed by Ho et al. (2013), which asymptotically converges in probability to the Riemannian mean expectation. OPS is efficient in practice since it avoids the calibration stage and repeatedly recalculating the Riemannian mean of the previous data. Figure 1 shows the pipelines of our methods. The aligned time series are given by

(11) $$\begin{array}{r} {Ê}_{i+1}=\frac{1}{N_{s}}M_{i+1}^{-1/2}E_{i+1} \end{array}$$

### 2.6. Experiments

Within-subject, cross-subject, and cross-dataset experiments were carried out in this work. In the within-subject experiment, we compared the subject-specific performance of both traditional methods and deep learning models. In the cross-subject experiment, the unsupervised transfer ability of two deep learning models was verified in a single dataset. In the cross-dataset experiment, we further validated deep learning models on different datasets with/without online re-centering transformation. The Wilcoxon signed rank test was used to compare the performance of different methods.

In the within-subject experiment, a Linear Discriminant Analysis classifier was used for CSP and FBCSP feature extraction methods. In CSP method, the number of selected spatial filters was set to two. The filter bank of FBCSP is 4–9, 8–15, and 15–30 Hz. The number of selected spatial filters in FBCSP was set to four. Both traditional algorithms and deep learning models were trained on the training and validation sets for each subject. In the cross-subject experiment, leave-one-subject-out cross-validation was carried out on each dataset. One subject was chosen as a test subject, and deep learning models were trained on the rest of subjects in the same dataset. In cross-dataset experiment, deep learning models were trained on all subjects of one dataset while the rest of datasets were both test datasets.

Architectures of Shallow ConvNet and EEGNet in experiments are listed in Tables 3, 4, respectively. Parameters of models were mainly from original papers (Schirrmeister et al., 2017; Lawhern et al., 2018) but were adjusted to fit our input size and sampling rate of data. The dropout probability was set to 0.5. The optimizer was Adam with learning rate set to 0.001. The batch size was 16 in within-subject experiment due to the limited number of available trials. In cross-subject and cross-dataset experiments, the batch size was 128. Instead of early stopping used in Schirrmeister et al. (2017), we trained for 120 epochs and selected the best model on validation set. Both models were implemented in PyTorch framework (Paszke et al., 2017).

## 3. Results

### 3.1. Within-Subject Classification Results

Within-subject classification accuracies of both traditional methods and deep learning models on eight datasets are listed in Table 5. Each method was tested under two conditions (with PS and without PS). Both methods achieved accuracies beyond the random level. The boldface in Table 5 shows that the accuracy of method with PS is higher than that without PS. The Wilcoxon signed rank test showed that the performance of EEGNet with PS was significantly better than that of EEGNet without PS (ShallowNet: *p* = 0.06; EEGNet: *p* = 0.008). No significant improvement was observed between traditional methods with PS and that without PS. In PhysionetMI and CBCIC2019004 datasets, the accuracies of deep learning models were lower than that of traditional methods.

**Table 5:** Within-subject Classification accuracies averaged on 10-folds.

	**CSP**		**FBCSP**		**MDRM**		**ShallowNet**		EEGNet^***^	
	**w/o PS**	**w/ PS**	**w/o PS**	**w/ PS**	**w/o PS**	**w/ PS**	**w/o PS**	**w/ PS**	**w/o PS**	**w/ PS**
BNCI2014001	0.68	0.66	0.70	**0.72**	0.68	0.68	0.76	**0.77**	0.78	**0.79**
BNCI2014004	0.70	0.69	0.74	**0.75**	0.69	0.69	0.79	0.79	0.79	**0.80**
PhysionetMI	0.56	0.56	0.59	**0.61**	0.57	0.57	0.53	**0.56**	0.51	**0.56**
Cho2017	0.57	0.57	0.60	0.59	0.58	0.58	0.68	0.68	0.65	**0.66**
Weibo2014	0.66	0.65	0.68	**0.69**	0.68	0.65	0.75	**0.76**	0.71	**0.74**
Zhou2016	0.81	**0.82**	0.89	0.88	0.80	**0.82**	0.83	**0.87**	0.84	**0.88**
CBCIC2019001	0.57	0.55	0.60	0.60	0.59	0.57	0.66	0.66	0.71	0.71
CBCIC2019004	0.69	0.69	0.74	0.73	0.70	0.70	0.65	0.65	0.62	**0.65**
Mean	0.65	0.65	0.69	**0.70**	0.66	0.66	0.71	**0.72**	0.70	**0.72**

Stars correspond to ^***^p < 0.01. The boldface shows that the accuracy of method with pre-alignment strategy (w/ PS) is higher than that without pre-alignment strategy (w/o PS).

**Figure 2:**
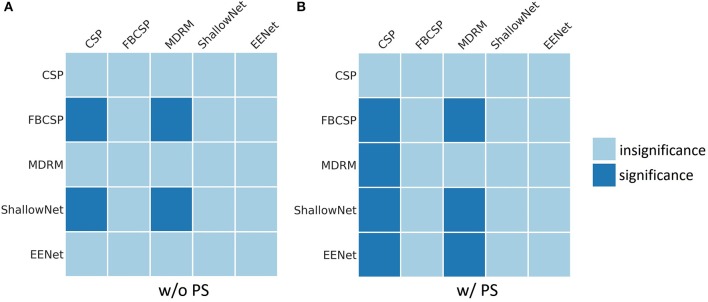
Results of the Wilcoxon signed rank tests on pairs of methods. The dark square shows that the performance of row method is significantly better than that of column method (*p* < 0.05). **(A)** Results of methods without pre-alignment strategy (w/o PS). **(B)** Results of methods with pre-alignment strategy (w/ PS).

Figure 2 shows results of the Wilcoxon signed rank test on each pair of methods. The dark square indicates that the performance of row method is significantly better than that of column method (*p* < 0.05). Under without PS condition, FBCSP and ShallowNet were significantly better than CSP and MDRM. Under with PS condition, all methods were significantly better than CSP. FBCSP, ShallowNet and EEGNet were significantly better than MDRM, whereas no significant differences were observed between deep learning models and FBCSP.

### 3.2. Cross-Subject Classification Results

**Figure 3:**
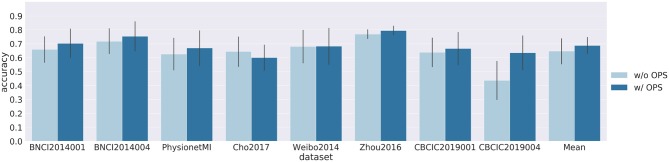
Results of cross-subject classification on eight datasets for ShallowNet with online pre-alignment strategy (w/ OPS) and without online pre-alignment strategy (w/o OPS). Leave-one-subject-out validation was implemented on each dataset, and the validation for each subject was repeated 10 times.

**Figure 4:**
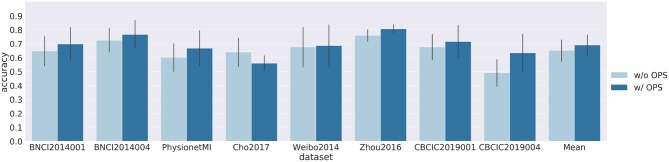
Results of cross-subject classification on eight datasets for EEGNet with online pre-alignment strategy (w/ OPS) and without online pre-alignment strategy (w/o OPS). Leave-one-subject-out validation was implemented on each dataset, and the validation for each subject was repeated 10 times.

Figures 3, 4 show results of cross-subject classification on eight datasets for ShallowNet and EEGNet, respectively. The performance of deep learning models without OPS in cross-subject classification was significantly higher than the random level (ShallowNet: *p* = 0.008; EEGNet: *p* = 0.008). ShallowNet with OPS was significantly better than that without OPS (ShallowNet: *p* = 0.046; EEGNet: *p* = 0.062). Specifically, for CNBCI2019004 dataset, ShallowNet with OPS increased the accuracy by 19.8% and EEGNet with OPS increased the accuracy by 14.3%. But for Cho2017 dataset, accuracies of models with OPS both suffered a little decrease (ShallowNet: 4%, EEGNet: 8%).

### 3.3. Cross-Dataset Classification Results

**Figure 5:**
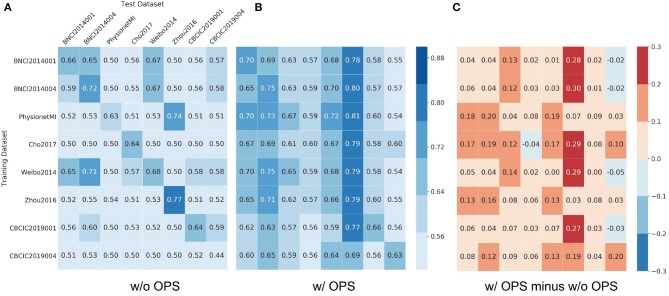
Results of cross-dataset classification for ShallowNet. The model was trained using the row dataset and validated on column datasets. In **(A)** Results of ShallowNet without online pre-alignment strategy (w/o OPS) and **(B)** Results of ShallowNet with online pre-alignment strategy (w/ OPS), the number in each square is the validation accuracy and the element of main diagonal is the cross-subject accuracy in each dataset showed in Figure 3. **(C)** The difference between **(B)** and **(A)**.

**Figure 6:**
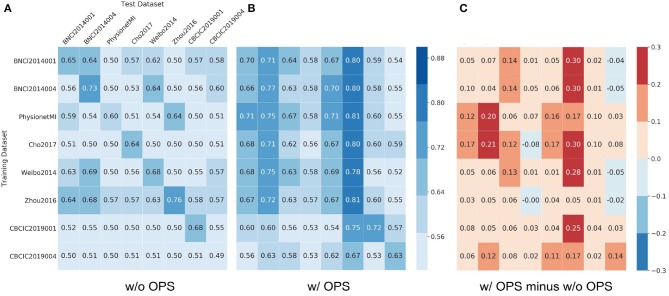
Results of cross-dataset classification for EEGNet. The model was trained using the row dataset and validated on column datasets. In **(A)** Results of EEGNet without online pre-alignment strategy (w/o OPS) and **(B)** Results of EEGNet with online pre-alignment strategy (w/ OPS), the number in each square is the validation accuracy and the element of main diagonal is the cross-subject accuracy in each dataset showed in Figure 4. **(C)** The difference between **(B)** and **(A)**.

Figures 5, 6 show results of cross-dataset classification for ShallowNet and EEGNet, respectively. The row label is the name of training dataset and the column label is the name of test dataset. The main diagonal element of each heatmap, where the training dataset is also the test dataset, is the cross-subject classification accuracy of the current dataset indicated in Figures 3, 4.

Figure 5A shows that most cross-dataset accuracies of ShallowNet without OPS were near the random level, although their within-dataset accuracies (elements of the main diagonal) were not. Figure 5B shows that cross-dataset accuracies with OPS were significantly more improved than that without OPS in all datasets (*p* < 0.05). Figure 5C shows the difference between Figure 5A and Figure 5B. Figure 6 shows similar results of EEGNet to that in Figure 5.

## 4. Discussion

To compare traditional methods and deep learning models, we first validated three traditional methods and two deep learning models in within-subject experiment. The results of within-subject experiment are listed in Table 5 and Figure 2. The performance of FBCSP was significantly better than that of CSP and MDRM while no significant differences were observed between deep learning models and FBCSP.

However, traditional methods are more robust in small sample learning. The performance of deep learning models is limited by the amount of data available. We observed that deep learning models were unable to achieve the same performance as traditional models in PhysionetMI and CBCIC2019004 datasets, which have relatively small number of trials per subject (PhysionetMI: 44; CBCIC2019004: 80). We also observed that pre-alignment strategy could significantly improve the performance of deep learning models while no significant improvement was found in traditional methods. The analysis of within-subject experiment indicates that deep learning models can achieve the same performance as traditional methods in subject-specific classification tasks with enough training data.

Our second analysis considered the feasibility of using deep learning models to solve cross-subject variability problems. Leave-one-subject-out cross-validation was carried out on each dataset. The results of the cross-subject experiment are shown in Figures 3, 4. The performance of deep learning models without OPS was significantly higher than the random level. The results indicate that deep learning models are able to transfer a pre-trained classifier to a new subject without additional subject-specific calibration data. We also tested deep learning models with OPS on eight datasets. Deep learning models with OPS were significantly better than those without OPS. The OPS aligns the data of each subject to the similar distribution, which makes deep learning models much easier to learn common patterns across subjects. We also noticed that Cho2017 dataset suffers performance lost in both deep models with OPS. This may due to different motor imagery instructions. The authors in Cho et al. (2017) asked subjects to imagine four sequential finger movements instead of the clench of fist in other datasets. Imagining finger movement, which is still an open problem, is much harder to decode than imagining fist clenching. Besides, we only used Cz, C3, and C4 channels to decode fist clenching imagery, which are not sufficient to decode finger movements. Using more channels around central area may improve the performance of Cho2017 since they can cover much larger motorsensory area.

Although deep learning models seem feasible in solving the cross-subject variability problem as depicted in Figures 3, 4, we note that deep learning models fail to generalize well in practice. Our third analysis explored the generalization ability of deep learning models on large datasets in the cross-dataset experiment. The results indicate that the cross-dataset variability problem reduces the generalization ability of deep learning models. In our second analysis, two models indeed have the ability to classify trials of a new subject without any calibration data in the same dataset. However, the pre-trained model in one dataset is unable to achieve the same performance on other datasets, which suggests that the model is highly specialized in its training dataset structure. Similar phenomenon was reported in paper (Jayaram and Barachant, 2018), where authors validated the use of traditional methods of different datasets in within-subject classification experiment. They found that the significance between algorithms depends on the specific dataset and results of a single dataset need to be tested on more datasets.

The reason for cross-dataset variability is still under exploring, but it may be caused by model overfitting problem. In cross-dataset classification scenario, a BCI dataset contains two kinds of variability: physiological variability and environmental variability. Physiological variability is responsible for the cross-subject variability while environmental variability is responsible for the environmental changes. Each dataset has its own specific configurations, including the amplifier, the electrode cap, the sampling rate, and the bandpass filtering settings. Moreover, data of subjects in the same dataset are acquired in the same laboratory environment. Deep learning models are usually trained on the data of all subjects of the same dataset. Since the distribution of environmental variability is more stable than that of physiological variability in the same dataset, deep learning models can easily overfit on the environmental variability. When the pre-trained model is validated on other datasets, which have different distributions of environmental variability, the model loses its generalization ability since the model is not robust to environmental changes.

One way to alleviate cross-dataset variability is to add more subjects from different datasets into the training set. However, cleaning data is hard due to different settings of public datasets. Instead of adding more subjects, we use an online pre-alignment strategy to reduce physiological variability of each subject without any calibration data. OPS significantly improves the generalization ability of deep learning models. Zhou2016 is the dataset with the most significant improvement. All models trained on other datasets can achieve more than 70% accuracy except for CBCIC2019004. The result is reasonable since Zhou2016 is a biased dataset in which all subjects are experienced subjects. We found that the classification accuracies for some datasets are even higher than their within-subject classification accuracies (comparing to FBCSP without PS). For example, for PhysionetMI, nearly all models trained on other datasets (except CBCIC2019001 and CBCIC2019004) can achieve more than 60% accuracy, which is higher than its within-subject accuracy (59%). This finding may suggest that deep learning models can extract more stable feature representation than traditional methods. We also found that different datasets have different impacts on deep learning model training process. The improvement of CNBCIC2019004 on other test datasets is limited compared to other training datasets. This may be due to one drawback of deep learning models. CBCIC2019004, which only has 480 trials totally, does not have enough data for training comparing to other datasets. In summary, we recommend two tips that may be helpful for deep learning based BCI research:

1. Use OPS as a preprocessing step.
2. Collect enough training data.

## 5. Conclusion

In this paper, we have validated deep learning models across eight MI datasets. The analysis shows that the cross-dataset variability would reduce the performance of deep learning models, suggesting the need of validating models on different datasets for future cross-subject studies. We also proposed the online pre-alignment strategy to improve generalization ability of deep learning models. The results demonstrate that deep learning models with OPS could achieve high performance for cross-subject classification without the calibration stage.

## Data Availability Statement

The datasets for this study, CBCIC2019001 and CBCIC2019004 can be found in the DataFountain website [https://www.datafountain.cn/competitions/342]. The remaining datasets for this study can be downloaded with MOABB package [https://github.com/NeuroTechX/moabb]. The source code for this study is available on request to the corresponding author.

## Author Contributions

All authors contributed to manuscript revision, and they read and approved the submitted version.

## Conflict of Interest

The authors declare that the research was conducted in the absence of any commercial or financial relationships that could be construed as a potential conflict of interest.
